# Developing Content for the Food Environment Assessment Survey Tool (FEAST): A Systematic Mixed Methods Study with People with Disabilities

**DOI:** 10.3390/ijerph17217781

**Published:** 2020-10-24

**Authors:** Rebecca E. Lee, Alicia O’Neal, Chelsea Cameron, Rosemary B. Hughes, Daniel P. O’Connor, Punam Ohri-Vachaspati, Michael Todd, Margaret A. Nosek

**Affiliations:** 1 Center for Health Promotion and Disease Prevention, Edson College of Nursing and Health Innovation, Arizona State University, 550 N. 3rd St., Phoenix, AZ 85004, USA; aloneal1@asu.edu (A.O.); chelsearose182@gmail.com (C.C.); 2 Population Health, University of Kansas School of Medicine, Kansas City, KS 66160, USA; 3 Rural Institute for Inclusive Communities, University of Montana, Missoula, MT 59812, USA; rhughes@ruralinstitute.umt.edu; 4 Department of Health and Human Performance, HEALTH Research Institute, University of Houston, Houston, TX 77204, USA; dpoconno@central.uh.edu; 5 College of Health Solutions, Arizona State University, Phoenix, AZ 85004, USA; Punam.Ohri-Vachaspati@asu.edu; 6 Edson College of Nursing and Health Innovation, Arizona State University, Phoenix, AZ 85004, USA; mike.todd@asu.edu; 7 Center for Research on Women with Disabilities, Department of Physical Medicine and Rehabilitation, Baylor College of Medicine, Houston, TX 77030, USA; mnosek@bcm.edu

**Keywords:** ecologic model of obesity, mobility impairment, disability, access, healthy eating

## Abstract

Almost 1 in every 8 adults in the U.S. have a physical disability that impairs mobility. This participatory project aimed to identify and describe environmental and personal barriers to healthy eating among people with mobility impairments using a rigorous, structured mixed methodology. Community-dwelling adults with a self-reported mobility impairment (*N* = 20, *M* = 40.4 years old, 60% female) participated in nominal group technique focus groups. The Ecologic Model of Obesity grounded stimulus questions asked about barriers to obtaining and preparing healthy food. Participants emphasized common barriers across everyday settings—focusing, for example, on the ability to reach shelved food inside the home, navigating to and inside stores and restaurants, and using delivery services. Home environments often did not afford suitable spaces for food preparation and storage. Participants reported inadequate transportation and numerous additional barriers in many settings to be able to eat healthfully. Participants reported lack of accessible transportation and architectural barriers inside stores, restaurants, and their own homes, highlighting the need for efforts aimed at improving accessibility and usability. Findings support the use of the Ecologic Model of Obesity to guide research and suggest the need for improvement in assessment practices and policies that enhance access to healthy food.

## 1. Introduction

Almost 43 million (about 1 in every 8) adults in the U.S. have a physical disability that impairs mobility [1]. The prevalence of people living and working with mobility impairments is increasing owing to technological and health innovations that improve survival and functioning after injury or with chronic disease [2]. People with mobility impairments also generally have inadequate nutrient intake, consume fewer fruits and vegetables, and are more likely to exceed daily recommendations for saturated fats than those without mobility impairments [3,4]. This contributes to increased mortality, morbidity, and risk for chronic conditions, and exacerbates disability-related limitations and other health problems [5,6,7,8].

Poor dietary habits are attributed, in part, to environmental and social barriers, such as lack of access to grocery stores and lack of support for healthy eating [9,10]. These barriers may operate differently among people with mobility impairments. Previous research with this population has focused primarily on older adults without support to leave their home or on those recently discharged from healthcare facilities [11,12,13,14,15]. This work has typically emphasized individual dietary assessment, and applied a deficits-based medical model, rather than an ecologic person-based approach. To date, little research has addressed the specific challenges and barriers affecting dietary habits for community-dwelling people with mobility impairments, and this gap is a significant impediment to understanding and mitigating the obesity disparities facing this population [16].

**Figure 1:**
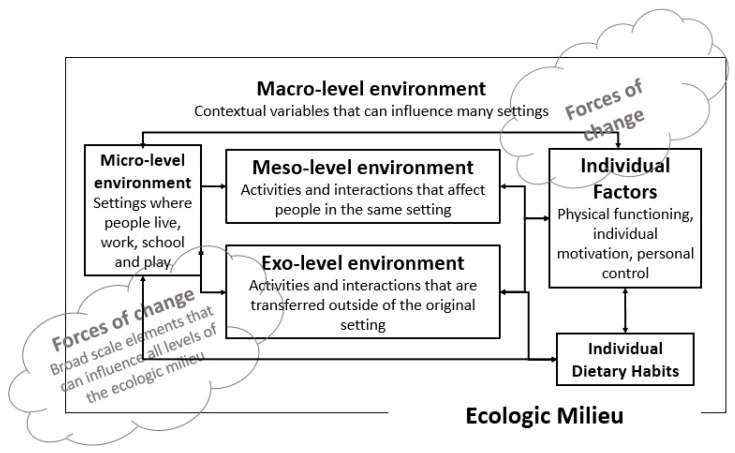
The Ecologic Model of Obesity adapted to investigate barriers to healthy dietary habits in people with mobility impairments.

The Ecologic Model of Obesity is a multilevel conceptual framework that encompasses physical environments, social and physical interactions, as well as intrapersonal mediators that operate as multi-directional, dynamic elements influencing dietary habits and weight status (see Figure 1) [17,18,19]. *Macro-level* policies can influence multiple settings and people. For example, the Americans with Disabilities Act (ADA) [20] guarantees equal opportunities in (for example) transportation, public accommodations such as in stores and restaurants, and telecommunication for people with disabilities. Policies at the macro-level like the ADA can foster equitable access to nutritious food. *Micro-level* environments comprise settings in which people live their day-to-day lives such as stores, restaurants, and the home, places where nutritious foods are commonly purchased, prepared, and consumed. *Meso*- and *exo-level* environments in the Ecologic Model of Obesity are dynamic, social and physical linkages between micro-level environment settings and people. These include access to support such as transportation and personal assistance both outside and within the home. *Individual factors* are physical or psychosocial influences on outcomes. Acting broadly across all levels within the *ecologic milieu* are *forces of change*, such as technological innovation and globalization. Although there has been a growing interest in research understanding how ecologic features influence dietary habits and weight, little attention has focused on people with mobility impairments, whose perspectives may be distinct from those of the general population [9,10].

The goal of this participatory research study was to define and describe barriers to healthy eating among people with mobility impairments using a rigorous, structured mixed methodology. We drew on the lived experience of community-dwelling urban and rural, women and men with mobility impairments. Identification of relevant barriers to healthy eating within a dynamic systems framework such as the Ecologic Model of Obesity can help inform the development and validation of a comprehensive instrument to measure ecologic and personal barriers to healthy dietary habits in people with mobility impairments. In turn, this can provide foci for future individual, environmental and policy interventions to improve healthy eating.

## 2. Materials and Methods

### 2.1. Participants

Men and women were eligible to participate if they reported (1) having a mobility impairment due to a chronic, physically disabling condition; (2) having the condition at least one year; (3) being free from cognitive, hearing, or visual impairment that would impede participation in the protocol; (4) being at least 18 years of age; (5) having access to and understanding how to use a working phone and/or an Internet-connected mobile device or personal computer with which they could receive email; and (6) residing in the United States. Participants were recruited to ensure representation by male or female gender and urban or rural location of residence. All study materials and protocols were approved by the Arizona State University Institutional Review Board.

### 2.2. Community Advisory Board

The Community Advisory Board (CAB) comprised five individuals with mobility impairments who had connections to organizations that served people with disabilities. Members included two men and three women, and came from five states—Minnesota, Arizona, Texas, Maine, and New Mexico. Project staff held quarterly (or more frequently if needed) Zoom video conferences with CAB members to review project materials. CAB members also reviewed materials offline and provided feedback as needed. Members each received $200 per year as long as they completed at least 75% of the 8 h agreed upon in memoranda of understanding.

### 2.3. Participant Recruitment

Recruitment was conducted by contacting organizations that served people with mobility impairments in urban and rural locations, outreach through the CAB, and via announcements posted on social media. Recruitment coordinators developed a database of 265 potential recruitment organizations and groups in rural and urban areas, including resource groups, durable medical equipment providers, private/public clinics and health care providers. Staff distributed electronic flyers promoting the study to these organizations to disseminate to their members. The flyers listed inclusion criteria, goals of the research study, participant incentives, and methods for receiving more information. Additional content included motivational messages and hashtags that were associated with the social media community of people with mobility impairments. The Food Environment Assessment Survey Tool (FEAST) project website (https://feastresearch.weebly.com) provided detailed information about the study, answered frequently asked questions, encouraged communication with a “contact us” form, and allowed participants to self-screen their eligibility to participate in the study. The CAB vetted all materials prior to use and provided feedback on the website.

Facebook, Twitter, and Instagram social media platforms were the primary recruitment tools. A FEAST Project account on each platform increased social awareness and assured credibility of the research study. Two to three times each week, recruitment coordinators posted from each account about the study and sought “friends” and followers to develop a larger network and recruitment pool.

Staff initiated private direct messaging contacts with approximately 700 Facebook, Twitter, and Instagram accounts. This resulted in the addition of 38 friends on Facebook and 241 followers on Instagram. Staff added the FEAST Facebook page to 15 disability-related groups and posted regularly in each group to initiate engagement with possible participants. Eighty individuals expressed interest in participating, and 14 of these participated in a nominal group technique (NGT) group or cognitive interview. When a social media account user responded expressing interest in participating, staff entered their information into a password-protected Excel spreadsheet. Recruitment coordinators followed up with account users until they either agreed or explicitly declined to participate in the study. It typically required multiple requests to obtain confirmation.

### 2.4. Nominal Group Technique (NGT) Groups

The investigative team drafted NGT prompts to stimulate responses about grocery shopping, food delivery services, meal planning, food storage, and dining at restaurants, focusing on the social and environmental barriers within and between settings, external to individuals. The investigative team and the CAB reviewed and revised prompts multiple times. The final NGT guide included seven prompts. The complete list of prompts is in Table 1.

NGT is an established, multistep group data collection procedure in which a group generates, collects, and prioritizes responses in reaction to a specified concept via a prompt. NGT produces unambiguous data, reduces group bias, is more efficient than traditional focus groups, and helps to contribute to unbiased content generation. NGT groups produce qualitative (emergent themes) and quantitative (ordinally ranked) data reflecting prioritized views about a specific concept [21,22]. To increase robustness, breadth, and representativeness of responses, the project conducted one NGT session with each of seven groups representing four pre-specified demographic strata: women in rural locations (2 groups), men in a rural location (1 group), women in urban locations (2 groups), and men from urban locations (2 groups).

NGT groups responded to the questions following a clearly defined protocol and script. Most groups were unable to complete all questions, so facilitators alternated questions to allow at least one of each of the four types of group to answer each question (see Table 1 for the number of groups answering each question). Two facilitators conducted each NGT session, one who read questions, instructions, and responses, and one who kept time and typed information into the shared screen using the Zoom platform. They presented each open-ended question on the shared screen and read it aloud twice to the group. The group had two minutes of silence to list as many responses as they could. The facilitators then used a round-robin approach, asking each participant to state a single response. The other facilitator typed each response on the shared electronic screen, visible to all participants. This continued until there were no new responses. Next, facilitators led a discussion to refine and clarify, but not criticize or evaluate, all responses. Participants determined together whether each response represented a single idea or needed to be broken into multiple ideas. They also had the opportunity to combine ideas that seemed to represent the same concept. NGT group sessions lasted about 90 min.

After the NGT group process was completed, a follow up survey listing all presented ideas was sent to participants of that group via email. Participants independently selected three to five responses that they believed were the most important for each probe. Staff combined these top selected responses from all participants in that group. Each participant received a $25 Amazon electronic gift card.

All FEAST study procedures were conducted in accordance with the ethical standards of the institutional and/or national research committee and with the 1964 Helsinki declaration and its later amendments or comparable ethical standards (Code: STUDY00008696). Informed consent was obtained from all individual participants included in the study.

### 2.5. Analysis

Responses about the same prompt from all groups that responded to it were combined into a single document. The investigative team examined this document and using a mixture of qualitative thematic analysis and quantitative frequency calculations to identify the most commonly mentioned themes.

Coders received training on thematic analysis. Coders first completed readings, and then practiced coding responses from one prompt. Two trained coders and one expert (REL) reviewed the NGT transcripts for each prompt independently to define emergent codes. Coders agreed upon and defined themes during the consensus coding process using a constant comparison approach [23]. When conflicting codes appeared, coders discussed their decisions and rationales until they reach consensus on an appropriate code. Codes that were linked closely in meaning were grouped into categories, and categories that were linked closely in meaning were, in turn, grouped into themes. A second review by one coder compared and contrasted themes. Once finished, the coders cross-checked and consolidated all coding. A third review of transcripts ensured that codes had reached saturation within the responses and that there were no additional emergent themes. Themes were then presented to the CAB for final comments.

## 3. Results

Twenty participants were allocated to seven NGT groups, representing urban women, urban men, rural women, and rural men. Descriptive information is presented in Table 2. One participant did not complete the demographic survey. Participants were young to mid-life adults, over half of whom were women, reflecting the study design. The majority of participants were white, born in the US, and spoke English at home. Over half had graduated college, and just under half reported that their parents were college graduates. Participants represented a diversity of incomes and living arrangements, both as singletons and shared housing. Participants represented 12 different states, residing in ZIP Code areas with a median of 2487 residents per square mile (range = 25–17,028 people per square mile). There were no significant differences on sociodemographic variables by urban or rural group assignment.

### 3.1. Shopping

Six of the seven groups answered the question about getting to and from the grocery store. Participants mentioned that getting to the store or a restaurant often depended on available transportation. One participant noted that, “Trying to find a ride getting to and help bringing the groceries home from the store,” was a substantial consideration, and this was universally endorsed as a top barrier within that group. Other participants noted there were limits to what one could carry, either because of one’s abilities to manage more than one thing at a time or items being too heavy or awkward to manage. Some participants mentioned transit rules limiting the number of packages per trip.

Bad weather was another barrier identified by participants as impeding grocery shopping. A participant in another group gave more details stating that it was, “Too hot driving in my car, and bad weather affects my health and ability to get to the store.” A participant who used a wheel chair remarked that, “Bad weather makes it difficult for me to roll to my grocery store. If nobody is around to drive me and the weather is bad, I can’t go.” Bad weather, arguably an annoyance for most people, seemed to create an additional layer of complexity for our participants.

Many participants also noted physical barriers to access inside grocery stores. The limits on what one could carry was also noted by several groups as an issue that was a barrier to shopping while inside the store. Many reported the concern of *navigation* within stores that did not accommodate people using assistive devices. For example, most groups (*n* = 4) raised the issues of aisles being too narrow or cluttered. One participant remarked, and all in that group agreed that, “(They) put displays in the middle of the aisle that are so hard to get around when riding in a mobility cart.” Participants who completed this question also universally (*n* = 6 groups) noted the *inability to reach things*, because counters or shelves were too high or low. One participant noted (and all participants in that group endorsed), “When I am inside the store the biggest issue that I have is not being able to reach things, shelves too high, or fruit crate that I can’t reach or inside a freezer.”

Most (4 of 6) groups who discussed barriers inside of grocery stores also identified *checkout* as problematic. Reasons include lanes being too narrow, counters at the wrong height, and stationary but unreachable credit card readers. As noted by one participant, “When you are paying with the card, the little machine is too high, and you can’t get it adjusted to the right height/scale to be able to use it.” Self-checkout was specifically mentioned as a common frustration. A participant in another group stated that there are “not enough checkout lines with actual people. Others are self-checkouts [and hard to use].”

In summary, participants identified transportation-related challenges and weather as important barriers to shopping. They also identified physical barriers inside stores, limits on what one could carry while shopping, and various features of checkout lanes as challenging during grocery shopping.

### 3.2. Restaurants

Reflections on experiences in restaurants tended to yield three consistent considerations of *navigation*, *inadequate seating* and lack of accessible *restrooms*. The majority in all groups that were asked this question (*n* = 5) mentioned and endorsed these three issues. Navigation within restaurants seemed problematic for all participants, regardless of whether they used wheelchairs or some other assistive device (e.g., walker, cane). For example, one participant stated, “Maneuvering the tables/chairs very difficult with my cane. Constantly getting it caught and tripping on it”. A person in another group stated that, “There might not be enough space between tables and chairs, I have to ask customers to move, possibly ask them twice”. “Large crowds and limited space”, were universal barriers for eating in restaurants for those with mobility impairments.

In addition to posing challenges with navigation, the internal seating arrangement of many restaurants does not sufficiently accommodate people with mobility impairments, especially those using wheelchairs and other assistive equipment. All groups indicated that the table height was too high or too low. All groups also discussed seating options that were never quite adequate, whether the issue was chairs being too low or too high, too small or simply not having enough. Inadequate seating is another universal barrier experienced in most restaurants.

Perhaps the most distressing finding was that all but one group raised the issue of “Lack of accessible restrooms once inside of the restaurant,” as one participant stated. Every group endorsed this as an important barrier to restaurant dining. A participant in one group emphasized, “Sometimes the bathrooms are inaccessible, I might not be able to close the stall door, the hallway might be blocked”. As well as the entrance to the restroom itself being a concern, a participant in another group stated that, “Sometimes the bathrooms in the restaurants are difficult to move around in”, suggesting that the interior of the restroom is also often problematic.

Perhaps reflecting ADA regulations implemented some three decades ago, only two of the groups mentioned, “Lack of an accessible entrance”, to the restaurant itself as one participant succinctly stated. A participant in a different group noted, “There might be a step to get in, or a damaged old ramp to get in”. The restaurant entrance was not highly endorsed, even when it was mentioned, suggesting that this may be a less commonly experienced barrier by people with mobility impairments.

In summary, participants noted challenges inside restaurants, especially with navigation around tables, chairs and other obstacles; seating arrangements and options that were not adequate for people using assistive devices; and lack of accessible restrooms.

### 3.3. At Home

#### 3.3.1. Storing/Retrieving Food in the Home

An overarching theme that emerged across several groups was that kitchens were often not designed with accessibility in mind. When asked about storing and retrieving food in the home, many participants noted insufficient *storage space* or issues related to a lack of access to storage. For example, one participant told us, “I don’t have as much storage room as other people, because I use a wheel chair, so I can’t use any storage that is up high. So, I run out of storage room”. Five out of six groups mentioned the specific issue of, “Not enough storage space…”, and all participants in those groups endorsed this as a top barrier. An issue connected to insufficient storage was the *inability to reach things* in the storage space that was available. One participant told us, “I can’t reach a lot of things in cupboards or on the shelf.” Every group that discussed this question mentioned this barrier and a range of unreachable storage spaces including shelves, cabinets, refrigerators, and freezers.

Participants in two groups mentioned *limits to what one could carry* or the ability to manage more than one thing at a time serving as a barrier, “Moving items, I can only carry one item at a time, difficult to carry multiple things at one time”. In some cases, this may have been related to the issue of products being *too heavy to lift or carry*. Three groups specifically discussed the weight of objects, for example, “Canned foods are sometimes too heavy to bring down from the shelves.” A third commonly mentioned problem was the fact that *opening and closing containers* could be challenging. For example, one participant stated, “Tupperware containers and anything with a lid could be difficult to open”.

In summary, research participants discussed how most home kitchens were not designed with accessibility in mind, with insufficient storage space that was often not accessible. They also noted that food was often stored in ways that were awkward or too heavy to carry and hard to open.

#### 3.3.2. Planning Meals or Snacks

Six groups responded to the question about planning meals or snacks. *Inability to reach things* was again a commonly identified issue, with four of the groups mentioning that items needed for a meal or snack were “out of reach”. This was a top issue in three of these four groups. One participant stated that it was difficult to plan without, “Knowing what I have in inventory at home already or do I need to get it. I can’t access the cupboard to determine what is in the cupboard”. Other groups echoed this sentiment, particularly in the context of having housemates who put things out of reach and out of sight for the person with the mobility impairment.

Beyond the barrier of not being able to reach inventory, participants mentioned the problem with general *limited inventory* altogether. This was a top choice in three of the four groups who mentioned it. Two subthemes of this construct emerged. The first, “*getting the groceries*”, was the biggest barrier to planning mentioned in one group. A member of another group explained, “You might have a recipe that calls for ingredients that you don’t typically use. Items may expire before you can use them again”, which narrowed her ability to plan more interesting healthy foods. A second subtheme was *cost of inventory*, and this was mentioned in three groups and a top choice in two of them. A participant in one group said that, “Healthy options are more expensive, so financially it may not be feasible to purchase what’s good for you”.

Given the barriers already cited, it was not surprising that four groups acknowledged that *planning is difficult*, for a variety of reasons, although this was not a top barrier for any of them. One participant simply stated, “Planning meals takes a lot of effort. It’s tiring”. One participant in another group acknowledged that, “It is hard to stay disciplined enough to focus on planning what I need to do”.

Mobility in the kitchen, and lack of an *appropriately designed cooking space* also came out of the responses to this question in about half of the groups, but the ranking was not consistent. For example, one participant stated that, “mobility around obstacles in the kitchen, having enough room to maneuver around the kitchen”, impeded planning. A member of another group mentioned that, “I only have a hot plate, and a size small refrigerator, and minimal cooking appliances”, which limited the kind of meal planning that was possible. Another participant in a different group added, “lack of room or space is a barrier. I have to be very specific in planning, because things don’t fit”.

In summary, participants mentioned that having difficulty reaching or seeing food inventory inhibited their ability to plan meals and snacks. Participants also mentioned having limited inventory kept in house as well as the complexities of planning being overwhelming and tiring.

#### 3.3.3. Preparing Foods

Four groups answered the question about barriers to preparing foods. Across the board, not having *appropriately designed cooking space* was a consistent and pervasive theme with all participants agreeing unanimously that their kitchens were just not designed for them to cook easily. All cited a lack of space, with one participant stating that, “it is difficult to move around in the kitchen”. Kitchens also didn’t seem to be the correct size, with things at the wrong height for easy access, such as, “the counters might be too high up.” A participant in another group poignantly illustrated this concern, by telling the group, “I have an average size stove but a lower chair, which means I burn my wrist on the edge of the pan when trying to stir using spatula. I get burned from the front burner”.

Three groups mentioned the common issue of *inability to reach things*, in particular with stove cook tops, and this was one of the most commonly endorsed items for each of these groups. For example, one participant noted, “Sometimes pots are too high up or too deep to see if the food is actually cooking”. A participant in another group raised the same issue, stating, “I can’t see the pots on top of the stove, can’t monitor the pan ingredients.”

A final thread that emerged in three of the groups and endorsed as a top choice two out of the three times it emerged, seemed to harken to the notion of the *kitchen as the center of hearth and home*, with cooking viewed as an activity done with other household members. For example, one participant mentioned, “Cooking together in a small space is fun, get to spend time together”. In contrast, another participant in another group seemed to bemoan the limitations of the available cooking space in stating, “It is difficult to cook with other people, chair is in the way, cook alone or prep food outside of the kitchen”. Cooking time seemed to be an activity that is undertaken collectively perhaps as part of tradition and ritual.

In summary, participants discussed how their kitchen cooking space was not designed well for cooking for them, and that they perceived cooking as an activity often undertaken with family or housemates.

### 3.4. Food Delivery Services

Five groups answered the question about food delivery services. Perhaps the most commonly mentioned issue in all five groups had to do with simply *receiving the delivery*. One participant noted, and all in that group endorsed, that merely, “Signing for your purchase at the door”, was a significant barrier. A participant in another group noted, “I can’t get to the door quickly, so sometimes the delivery people don’t think I am going to come to answer the door”. This may seem to remedy these issues, but the lack of personal attention may also be more challenging, as one participant in another group stated, “Accepting the food at the door is difficult, because of the weight and quantity”. This might also be challenging in cases, when “…delivery services leave it out front where I’m unable to get it in time and the food might go bad.”

Another commonly mentioned barrier in four of the five groups, and endorsed as most important by two of them, focused on *food packaging*, either being unwieldly or otherwise hard to manage or open. Delivered food typically comes in secure packages to minimize the number of parcels and to withstand transport from the store or restaurant. Thus, as alluded to above in the concerns about receiving the delivery at the door, a participant in one group noted a similar issue of, “The groceries bags and boxes might be too heavy”. Other groups discussed this same issue. One participant noted that, “Ordering food from restaurants, the type of containers might be hard to open or access the food”, suggesting that packaging for ease of delivery did not lead to ease of consumption.

Other issues mentioned by the groups included concerns about cost, lack of service availability, unavailability of specific items, and slowness or timing of deliveries, which are all *supply-and demand-related logistics* that affect all users, regardless of mobility impairments. Concerns about cost were cited in three groups and ranked highest in all three. One participant noted, “For people that are on fixed incomes, some places add a premium; it is more expensive to have it delivered than just to go to the store”. Availability of service was noted in four groups, but usually endorsed by only one participant in the group. Issues about the availability of specific items such as, “the order is sometimes wrong”, or “unable to return items if they are wrong”, came up in three groups and was the highest endorsed item in two. Respondents in two groups, one rating it most highly, noted that time to delivery was slow, “Not quick turn-around for online orders, it could take possibly 24 h to a few hours”. These common complaints about food delivery may be resolved as availability of these services becomes more widespread.

In summary, participants discussed challenges with food delivery such as the difficulty in taking receipt of delivered food, carrying and opening packages, and logistical issues such as cost, availability and incomplete or incorrect items in the delivery.

## 4. Discussion

The purpose of this study was to identify and describe barriers to healthy eating among people with mobility impairments by applying a mixed methods approach, guided by the Ecologic Model of Obesity [19]. We found common themes across settings that described barriers focusing on navigating environments and the ability to reach things in stores, restaurants and at home. Home environments often did not afford suitable cooking and storage space for many participants. We found complaints of inadequate support in existing transportation networks and a need for help in many settings as barriers to eating healthfully.

Considered within the Ecologic Model of Obesity, commonly frequented neighborhood micro-level environments such as stores and restaurants often posed challenges related to navigation [19]. Participants did not mention concerns with navigating through neighborhoods, but focused almost exclusively on interior spaces. Although these settings are obliged to comply with policies that required adequate space for people using assistive devices, many participants reported violations. Our findings were consistent with previous research, that having adequate space, ease of entryways, and easily available amenities such as restrooms were essential for people with mobility-impairing disabilities [24]. We found navigation inside grocery stores and restaurants often thwarted by obstacles, which might not pose concerns for others. Partially blocked store or restaurant aisles are a design feature that could easily be remedied but seemed to have been overlooked. Our participants also reported an analogous concern with in-store presentation of goods. Goods may often be on high or low shelves or deep inside refrigerated cases. Stocking shelves vertically, rather than horizontally, might aid in the acquisition of dry goods; vertical merchandising may also increase store sales, making this a win-win strategy for consumers and merchants [25]. Counters can be too high for people who use wheelchairs. A final concern mentioned by participants involved restrooms, which, while often affording accessible entry, did not always offer accessible interior spaces that a person using an assistive device could use easily.

The home environment, the micro-level environment where people spend most of their time, also received generally poor marks from our participants. Participants focused on barriers related to things being out of their reach, having poorly designed cooking spaces in which to cook easily and safely, and many considerations focused on inventory and storage. Developing or modifying homes that are universally accessible may be an unattainable luxury for many people with disabilities who often have fewer financial resources compared to the general population [26,27]. Some states, however, have Medicaid waiver programs that may cover the cost of home modifications for eligible parties. Additionally, there are some national organizations that provide financial assistance to cover part or all of those costs.

In discussing barriers, most groups mentioned situations that served as barriers, where having help might have helped to overcome them. If this were the case, this would be consistent with our hypothesized meso- and exo-level environmental linkages from the Ecologic Model of Obesity [19]. For example, other studies have reported that helpers are vital to overcoming barriers reported by older adults with mobility impairments [24]. Many, if not all, of the barriers cited by our participants, e.g., reaching high or deep store shelves, being seen at the deli counter, pushing the cart, could be overcome by having a helper. This may also be true in restaurants, where participants reported inability to move furniture out of the way to get to the table. Participants noted barriers to using food delivery services, some specific to those with mobility impairments, and others more general. Of note, these data were collected prior to the COVID-19 pandemic and stay-at-home orders. Many delivery services have gone to “contact free” delivery, where there is no interaction with the delivery person and worked to improve the more general supply-and-demand issues.

Reliable transportation was another important exo-level environmental linkage between people and places. Given the regulations of the ADA and other policies to improve transportation, it is disconcerting that a lack of reliable transportation was mentioned so frequently. Other studies have reported that public transit is often an unreliable transportation strategy for this population, particularly in rural settings [28]. People with disabling mobility impairments may be unable to walk to transit stops, or have to wait for a ride with a friend [24]. For those who were able to use a private car, parking was a concern for many. The issue about not having enough accessible parking spots, people parking illegally in disabled parking spots, or insufficient space around the spots seemed to be common barriers for many.

Having personal control over one’s circumstances is an individual-level factor that can mediate or moderate environmental factors in the Ecologic Model of Obesity [19]. For example, since many of the meal planning barriers that participants identified seemed not about planning, per se, but more about actually doing things, like securing provisions and preparing them, may suggest that there is very little independent meal planning occurring, and little personal control. Considerations about personal safety such as with using a stove top and fatigue from the planning involved in eating healthfully may be individual-level limiting factors. Our findings suggest that meal planning, grocery shopping, and food preparation comprise an intertwined set of collective activities, which most participants have to consider, and which have to be considered by others in their household.

Responses suggested that participants perceived an inability to exercise the discipline needed to make healthy dietary choices, as evidenced in the responses to planning meals or snacks. They also suggested that cooking meals was part of a family or household endeavor. Perceived lack of personal discipline or “willpower” is often reported by all people trying to establish or maintain healthy habits that require exerting extra effort, particularly when there are others sharing meal planning, food shopping, and cooking responsibilities [14,24]. It may be impossible to overcome this perceived barrier in an unsupportive environment. When coupled with the additional layer of barriers faced by this community, this perceived lack of internal control may make it nearly impossible to maintain healthy dietary habits, highlighting the need for policy changes or increased financial support for more rigorous implementation of existing policy. Others have reported that not having access to healthy foods may serve as a significant barrier to optimal nutrition and healthy eating [9,29].

Another commonly cited barrier was bad weather, which operates as a macro-level factor, affecting all settings and residents in an area, not solely those with mobility impairments. However, using an assistive device may add to the complication beyond merely donning a jacket and heading out to accomplish one’s daily activities. Although bad weather can pose challenges to everyone, the challenges for people using wheelchairs or other assistive equipment to get from place to place can be exponentially worse, especially during winter, e.g., when snow and ice bring community participation to a standstill.

The findings from the current study provide rich information that authenticates theorized ecologic barriers to healthy eating from the perspective of people with mobility impairments. Strengths of the study include the use of a carefully constructed, theoretically-informed, a priori question guide; a robust systematic qualitative strategy and protocol for generating information; multiple well-trained group moderators; and experienced and well trained coders. Coders were able to compare and discuss themes with the community advisory board to improve reliability further [23]. Participants in this study were volunteer men and women representing urban and rural locations. The ability to judge the frequency and depth of participant views was a strength of this study, enhancing confidence in the findings. Participants were able to provide critical insight based on their unique perspective as members of the community. This was a qualitative inquiry, and thematic saturation was observed, enhancing confidence in the robustness of the findings; nevertheless, replication of these findings given the quantitative nature of some of the results, via additional research with larger samples is warranted. Given the dearth of information in this domain, these findings justify the development of theory and testing in this new area of inquiry.

Findings from this study support the use of the Ecologic Model of Obesity to guide research in the promotion of healthy eating among people with mobility impairments [19]. As presented in the results and Table 1, ecologic factors nominated by participants suggest future study. There is room for improvement in policies and practices that enhance access to healthy food, particularly as the community of people living and working with mobility impairments continues to grow as result of enhancements in medical science and technology. Consistent considerations of interior architectural deficits in micro-level environments such as stores, restaurants and homes evokes a need for re-thinking from design specialists. Improvements in exo- and meso-level factors such as help for shopping, planning and cooking are needed along with better transit.

Improved implementation and enforcement of existing macro-level policy is also needed. Additional research is indicated to test these findings systematically in larger samples using rigorous methodologies and analyses. Moreover, future research is needed to develop and implement training programs for grocery stores and other entities to reach compliance with existing disability regulations and to increase their sensitivity to people with disabilities (e.g., by training staff to assist disabled customers by retrieving merchandise from shelves and displays). Multiple efforts are needed to improve the profound lack of access that participants reported when trying to navigate the built environment in their own homes or inside grocery stores and restaurants. Full compliance with the ADA could eliminate many of the barriers that people with mobility impairments experience in accessing healthy food.

## Figures and Tables

**Table 1:** Descriptive summary of nominal group technique (NGT) questions, groups, themes and frequency mentioned.

Question	Groups	Themes	Frequency	EMO Level
Think about the last time you went to the grocery store. What were the physical barriers or problems that you experienced while getting to or coming home from the grocery store?	6	-Available transportation	5	Exo-level
		-Limits to what one could carry	6	Individual
		-Bad weather	5	Macro-level
Think about the last time you were physically inside a grocery store. What were the barriers or problems that you experienced inside the store? Think about getting around the store, finding what you needed, and purchasing your items?	6	-Navigation	4	Micro-level
		-Inability to reach things	6	Micro-level
		-Checkout	4	Micro-level
Think about the last time you ate out a restaurant. What makes eating out at a restaurant difficult?	5	-Navigation	5	Micro-level
		-Inadequate seating	5	Micro-level
		-Restrooms	5	Micro-level
Think about problems that you may encounter in storing food. What are the barriers to accessing, retrieving, and moving stored food?	6	-Storage space	5	Micro-level
		-Inability to reach things	6	Micro-level
		-Limits to what one could carry	2	Individual
		-Too heavy to lift or carry	3	Micro-level
		-Opening and closing containers	3	Micro-level
Tell me about barriers to planning meals or snacks? Think about relying on others or yourself to plan meals and snacks?	6	-Inability to reach things	4	Micro-level
		-Limited inventory		Micro-level
		-Getting the groceries	4	
		-Cost of inventory	3	
		-Planning is difficult	4	Individual
		-Appropriately designed cooking space	3	Micro-level
Now let’s talk about the barriers or problems you experience when you or your assistant prepares meals at home. Not including grocery shopping, what are some barriers or benefits you experience when preparing meals at home?	4	-Appropriately designed cooking space	4	Micro-level
		-Inability to reach things	3	Micro-level
		-Kitchen as the center of hearth and home	3	Meso-level
Many grocery stores have begun to offer delivery services. These services allow customers to choose their items online. Store employees will then shop for the items and deliver them to the customer. Food delivery companies like GrubHub, Postmates, and Uber Eats, also offer services to pick up the order and bring it to you. What do you think about these? What has your experience been?	5	-Receiving the delivery	5	Exo-level
		-Food packaging	4	Exo-level
		-Supply and demand related logistics		Force of Change
		-Cost	3	
		-Lack of availability of service	4	
		-Lack of availability of specific items	3	
		-Time to delivery	2	

Note. Frequency refers to the number of groups from the Groups column that nominated this issue as a barrier.

**Table 2:** Participant background characteristics.

Variable	*M* (*SD*) or % (*n*)
Age *M* (*SD*)	40.4 (16.3)
Number in Household *M* (*SD*)	2.3 (1.4)
Gender	
Female	33.3% (3)
Male	66.7% (6)
Race/Ethnicity	
African American	10.5% (2)
Native American	5.3% (1)
Latino/Hispanic	10.5% (2)
White	63.2% (12)
Biracial	10.5% (2)
Language spoken at home	
English	89.5% (17)
English/Spanish	10.5% (2)
Annual household income before taxes	
$0 to $25,000	37.0% (7)
$25,001 to $57,000	31.7% (6)
$57,001 or more	31.6% (6)
Educational attainment	
Some high school/High-school graduate (or test equivalent)	21.1% (4)
Some college or junior college	15.8% (3)
College graduate (from a 4-year college or university)	63.2% (12)
Parent educational attainment	
High-school graduate (or test equivalent)	42.1% (8)
Some college or junior college	5.3% (1)
College graduate (from a 4-year college or university)	47.4% (9)
Unknown	5.3% (1)
How did you hear about the FEAST study?	
Facebook	42.1% (8)
Instagram	15.8% (3)
FEAST Website	5.3% (1)
Other	36.8% (7)
